# Supplementary material for: Screening of EWI-2-Derived Peptides for Targeting Tetraspanin CD81 and Their Effect on Cancer Cell Migration
Source: Biomolecules. 2023 Mar 10;13(3):510. doi: 10.3390/biom13030510 (PMC10046862; doi:10.3390/biom13030510)
Supplement: Supplementary file 1 [file biomolecules-13-00510-s001.zip › biomolecules-2174233-supplementary.pdf]

Supporting information

Screening of EWI-2-Derived Peptides for Targeting Tetraspanin CD81 and Their Effect on Cancer Cell Migration

Thanawat Suwatthanarak [1,2,3], Kei Usuba [1], Kotomi Kuroha [1], Masayoshi Tanaka [1,4] and Mina Okochi [1,*]

1 Department of Chemical Science and Engineering, Tokyo Institute of Technology, 2-12-1-S1-24 O-okayama, Meguro-ku, Tokyo 152-8552, Japan

2 Siriraj Cancer Center, Faculty of Medicine Siriraj Hospital, Mahidol University, 2 Wanglang Road, Bangkok Noi, Bangkok 10700, Thailand

3 Department of Surgery, Faculty of Medicine Siriraj Hospital, Mahidol University, 2 Wanglang Road, Bangkok Noi, Bangkok 10700, Thailand

4 Department of Chemical Science and Engineering, Tokyo Institute of Technology, 4259 Nagatsuta-cho, Midori-ku, Yokohama-shi 226-8501, Kanagawa, Japan

* Correspondence: okochi.m.aa@m.titech.ac.jp

Table S1. Peptide number and sequence of 8-mer peptides constructed from the amino acid sequence of EWI-2 protein from the Ig3 domain to the C-terminus.

Peptide No.	Sequence	Peptide No.	Sequence	Peptide No.	Sequence
76	LSSQLAVT	102	RCLAKAYV	128	GVRPGGGP
77	LAVTVGPG	103	KAYVRGSG	129	GGGPVSVE
78	VGPGERRI	104	RGSGTRLR	130	VSVELVGP
79	ERRIGPGE	105	TRLREAAS	131	LVGPRSHR
80	GPGEPLEL	106	EAASARSR	132	RSHRLRLH
81	PLELLCNV	107	ARSRPLPV	133	LRLHSLGP
82	LCNVSGAL	108	PLPVHVRE	134	SLGPEDEG
83	SGALPPAG	109	HVREEGVV	135	EDEGVYHC
84	PPAGRHAA	110	EGVVLEAV	136	VYHCAPSA
85	RHAAYSVG	111	LEAVAWLA	137	APSAWVQH
86	YSVGWEMA	112	AWLAGGTV	138	WVQHADYS
87	WEMAPAGA	113	GGTVYRGE	139	ADYSWYQA
88	PAGAPGPG	114	YRGETASL	140	WYQAGSAR
89	PGPGRLVA	115	TASLLCNI	141	GSARSGPV
90	RLVAQLDT	116	LCNISVRG	142	SGPVTVYP
91	QLDTEGVG	117	SVRGGPPG	143	TVYPYMHA
92	EGVGSLGP	118	GPPGLRLA	144	YMHALDTL
93	SLGPGYEG	119	LRLAASWW	145	LDTLFVPL
94	GYEGRHIA	120	ASWWVERP	146	FVPLLVGT
95	RHIAMEKV	121	VERPEDGE	147	LVGTGVAL
96	MEKVASRT	122	EDGELSSV	148	GVALVTGA
97	ASRTYRLR	123	LSSVPAQL	149	VTGATVLG
98	YRLRLEAA	124	PAQLVGGV	150	TVLGTITC
99	LEAARPGD	125	VGGVGQDG	151	TITCCFMK
100	RPGDAGTY	126	GQDGVAEL	152	CFMKRLRK
101	AGTYRCLA	127	VAELGVRP	153	FMKRLRKR

Control sequences: Peptide number 154, AAAA; Peptide number 155, DDDD; Peptide number 156, RRRR; Peptide number 157, KKKK.

A

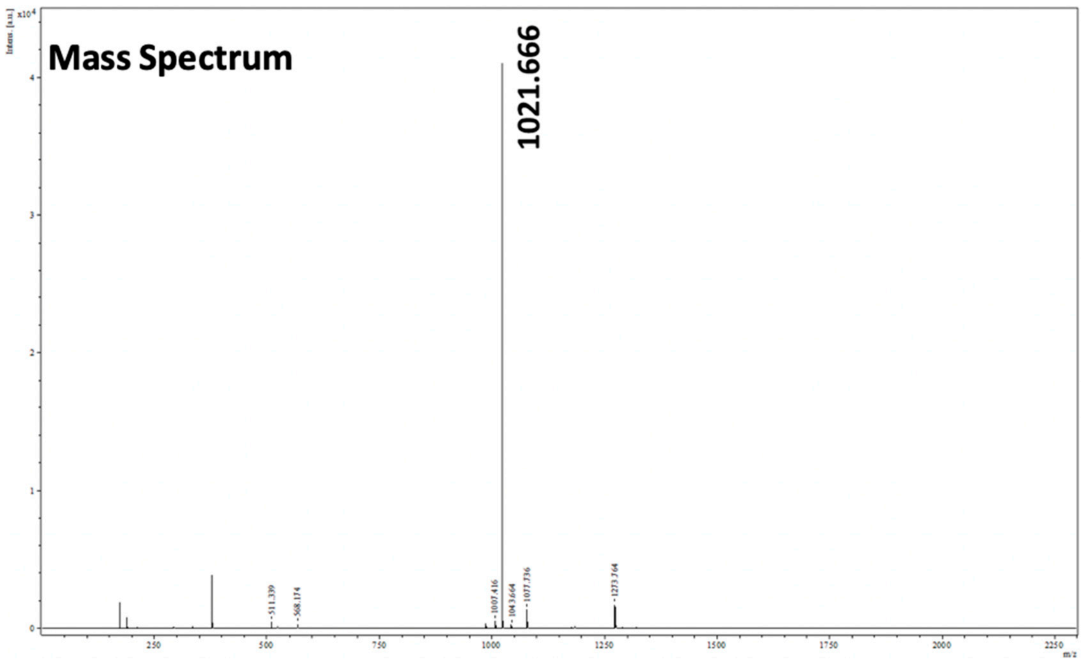

B

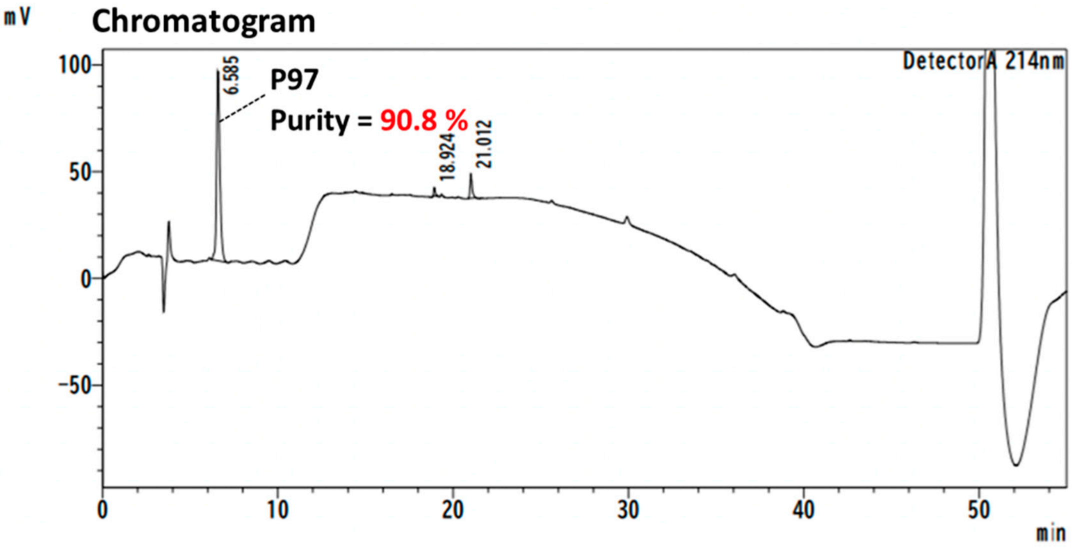

Figure S1. (A) MS spectrogram and (B) HPLC chromatogram of P97.

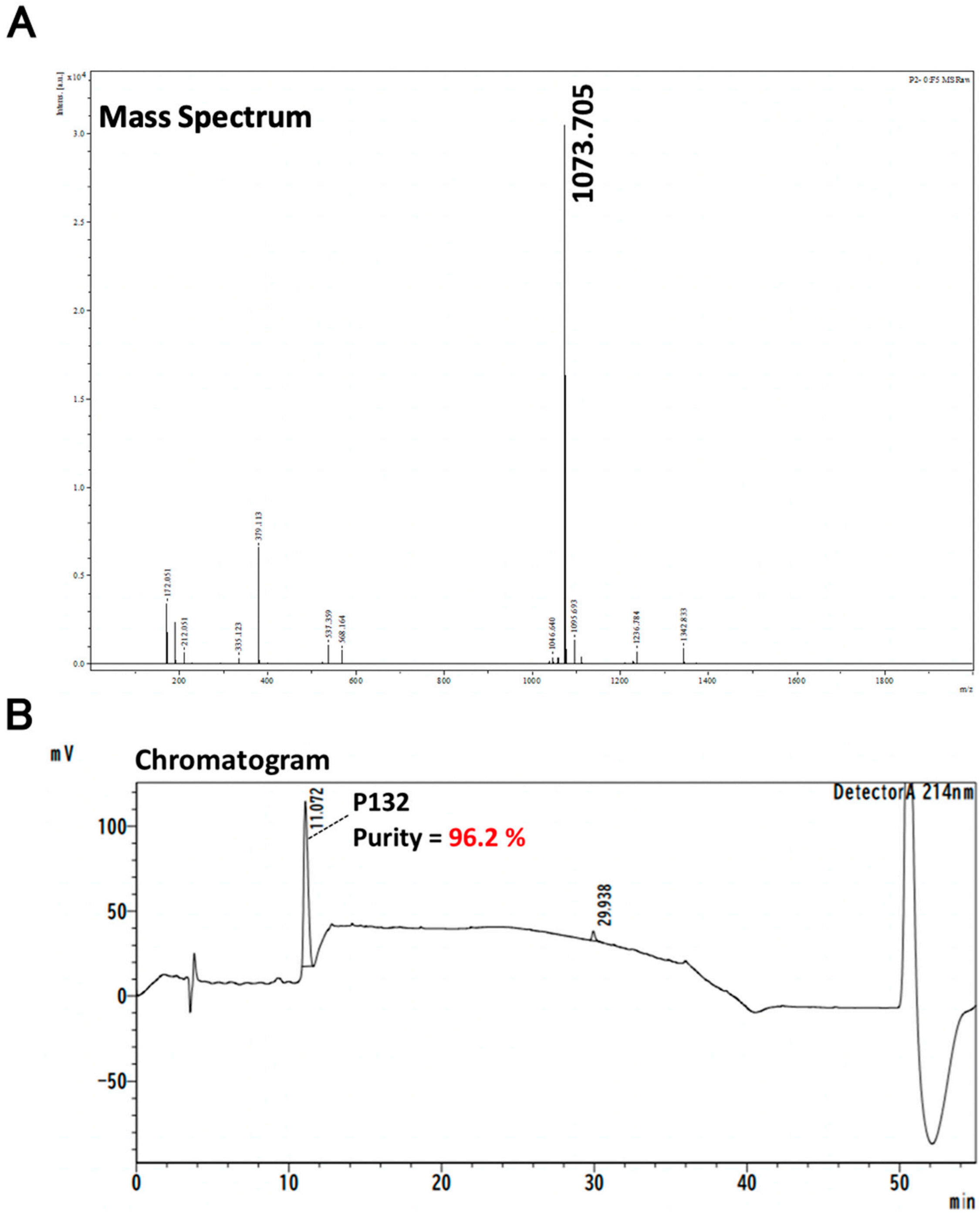

Figure S2. (A) MS spectrogram and (B) HPLC chromatogram of P132.

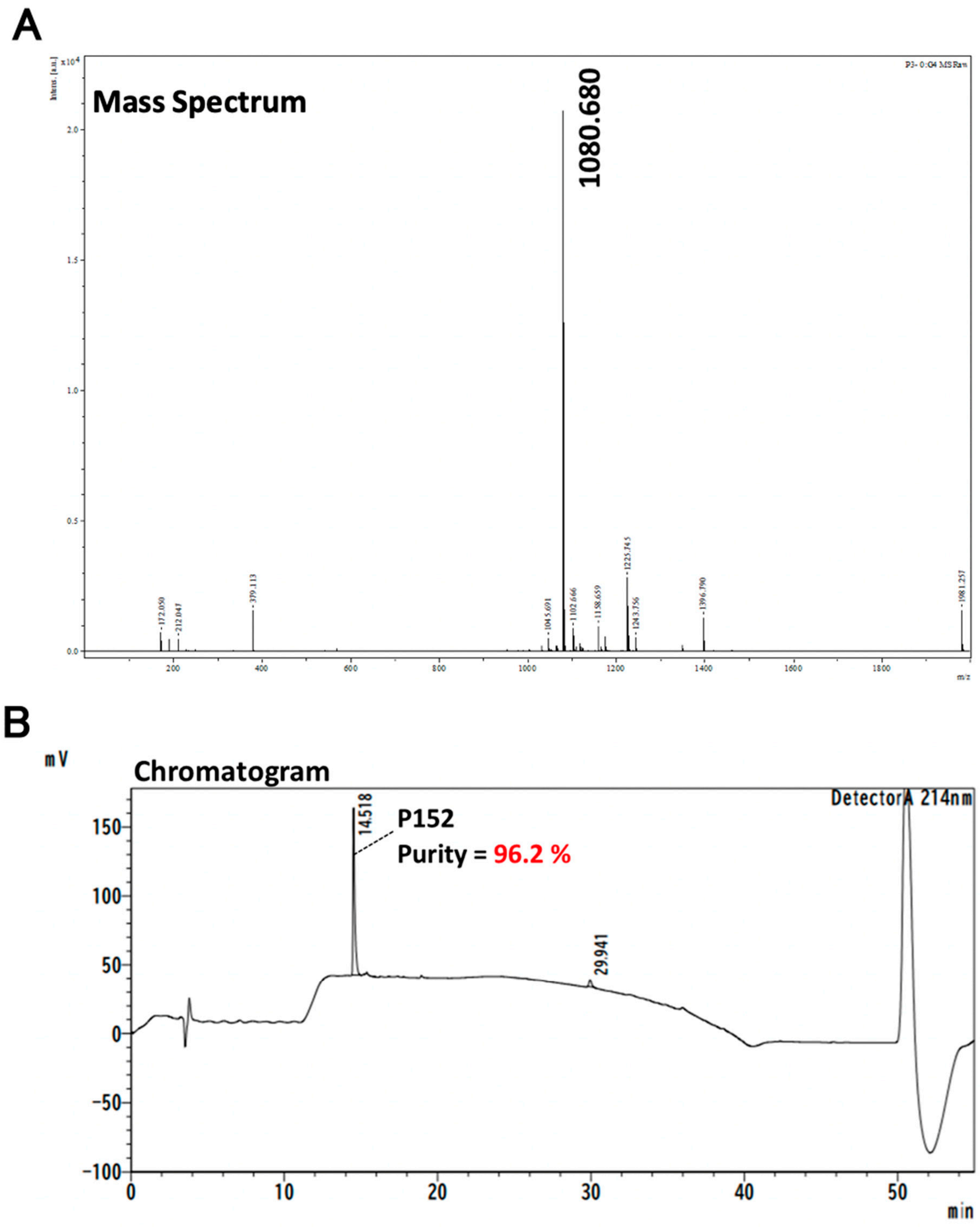

Figure S3. (A) MS spectrogram and (B) HPLC chromatogram of P152.

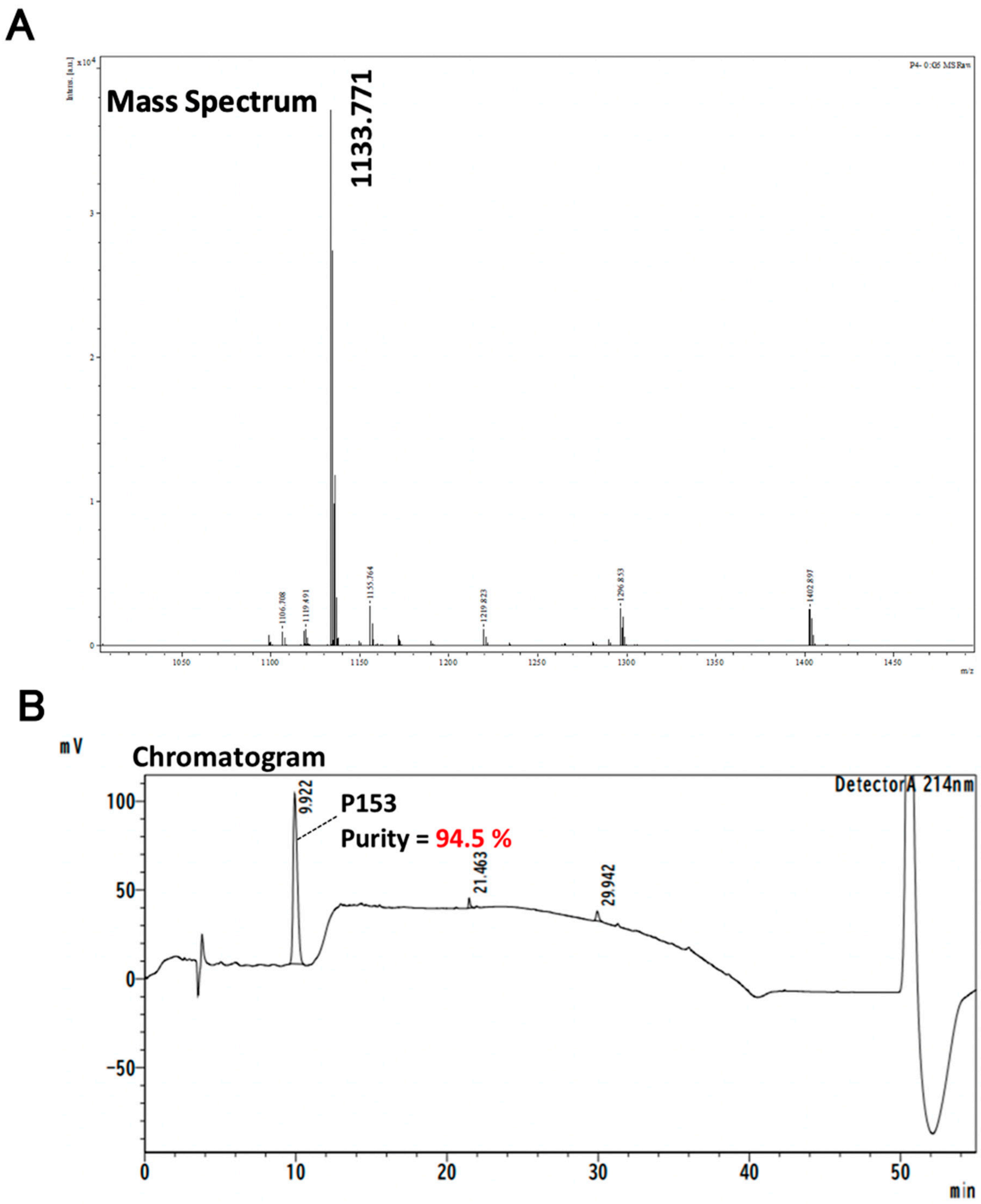

Figure S4. (A) MS spectrogram and (B) HPLC chromatogram of P153.

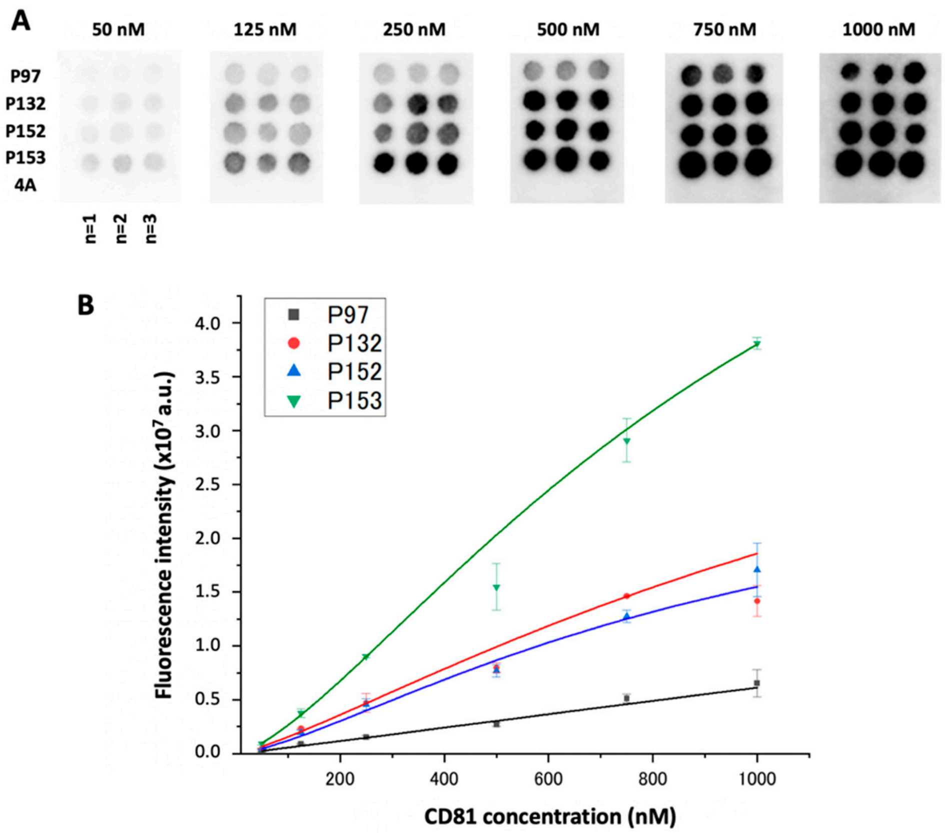

Figure S5. (A) Fluorescent images of peptide arrays containing CD81-BP candidates after binding assay with between Alexa Fluor® 488‒labeled CD81 protein at different concentrations. (B) Negative control (4A or AAAA peptide)-subtracted spot intensities of CD81-BP candidates against CD81 concentrations for estimating K_D values. Error bars show SD (n=3).

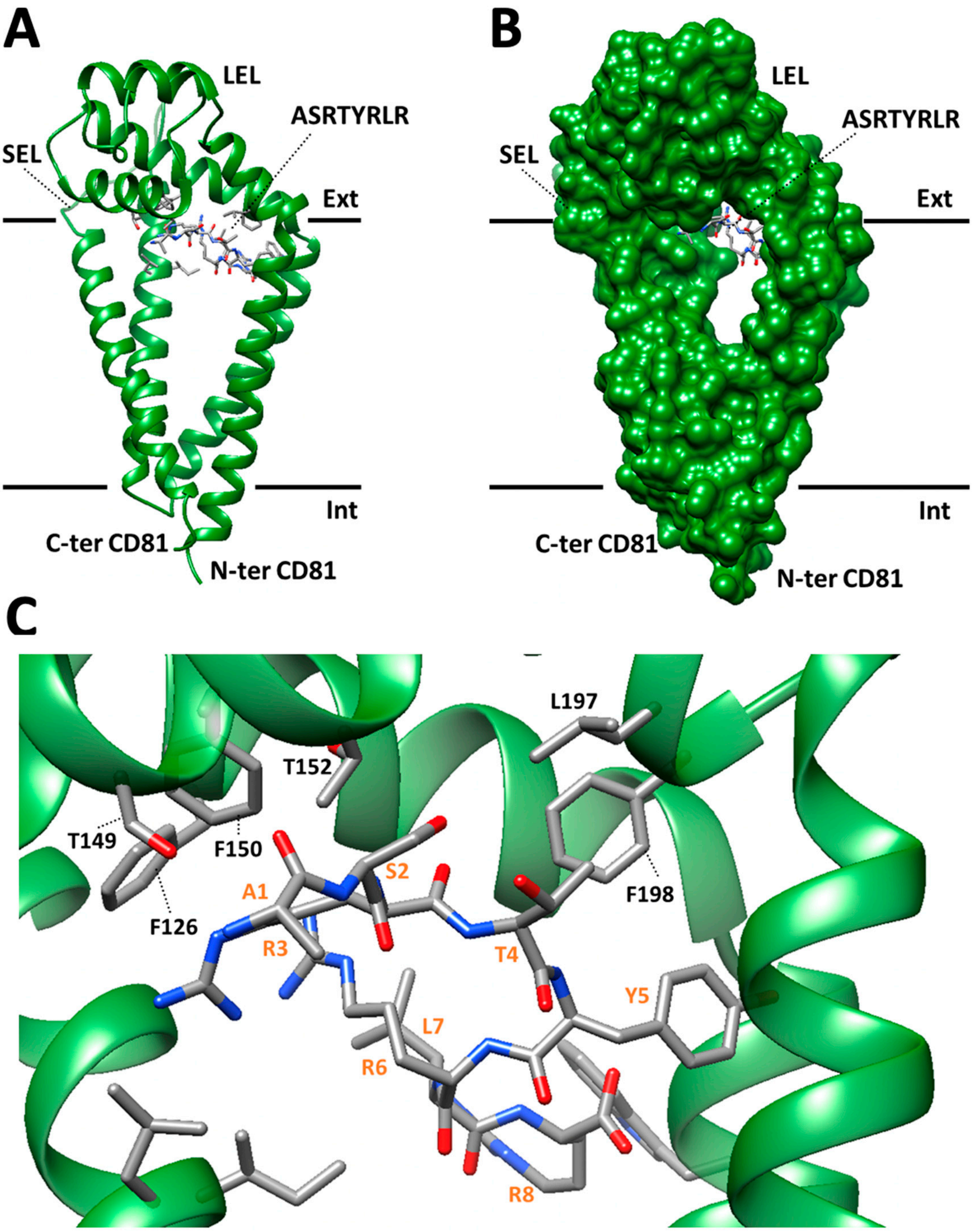

Figure S6. Protein−peptide complex structure of P97 and CD81 protein predicted by MDockPeP server [22]. (A) Protein−peptide complex structure of P97 and CD81 protein. (B) Surface representation of the molecular docking. (C) Zoomed view of the molecular docking. LEL, large extracellular loop. SEL, small extracellular loop. Ext, extracellular. Int, intracellular.

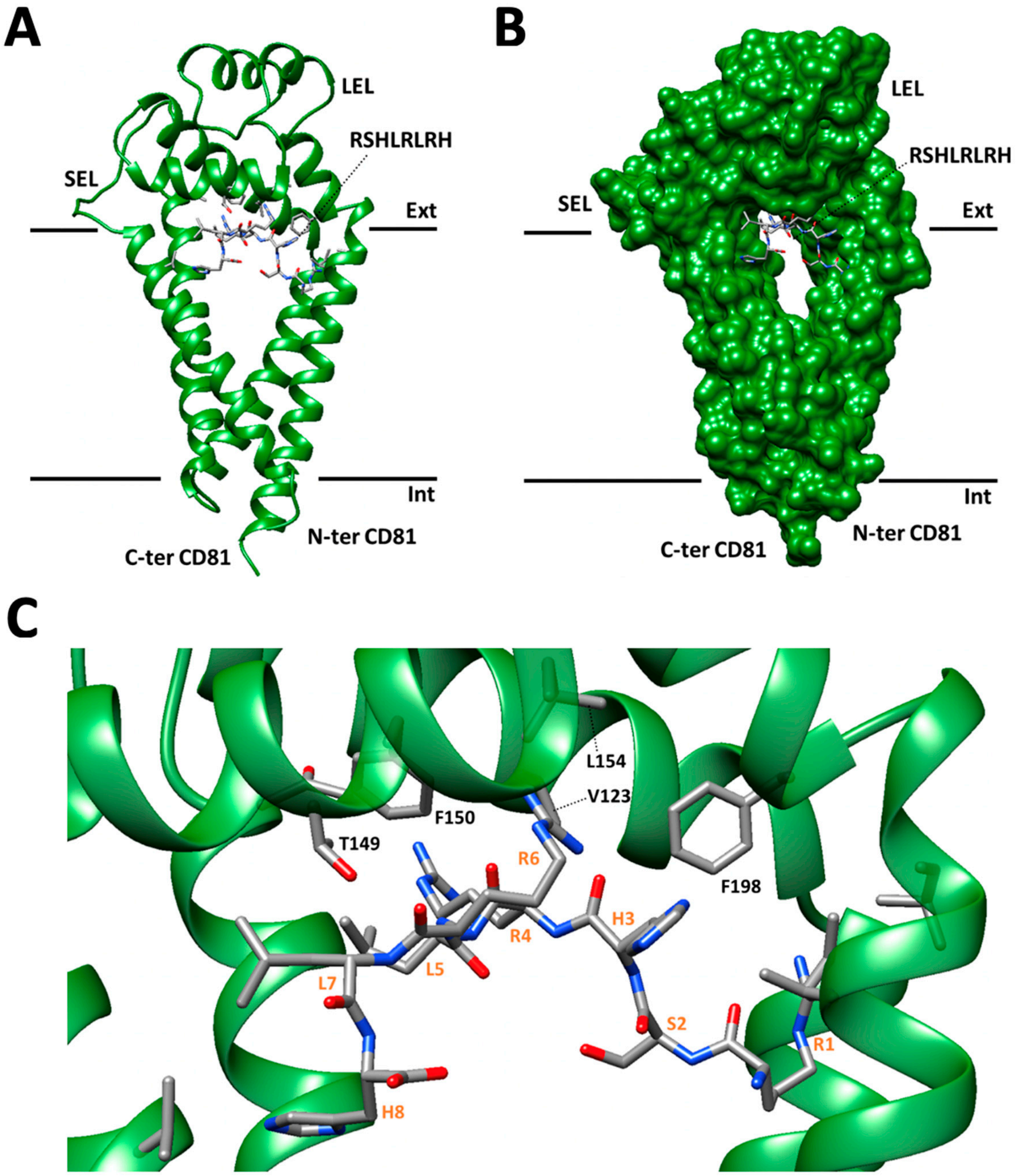

Figure S7. Protein−peptide complex structure of P132 and CD81 protein predicted by MDockPeP server [22]. (A) Protein−peptide complex structure of P132 and CD81 protein. (B) Surface representation of the molecular docking. (C) Zoomed view of the molecular docking. LEL, large extracellular loop. SEL, small extracellular loop. Ext, extracellular. Int, intracellular.

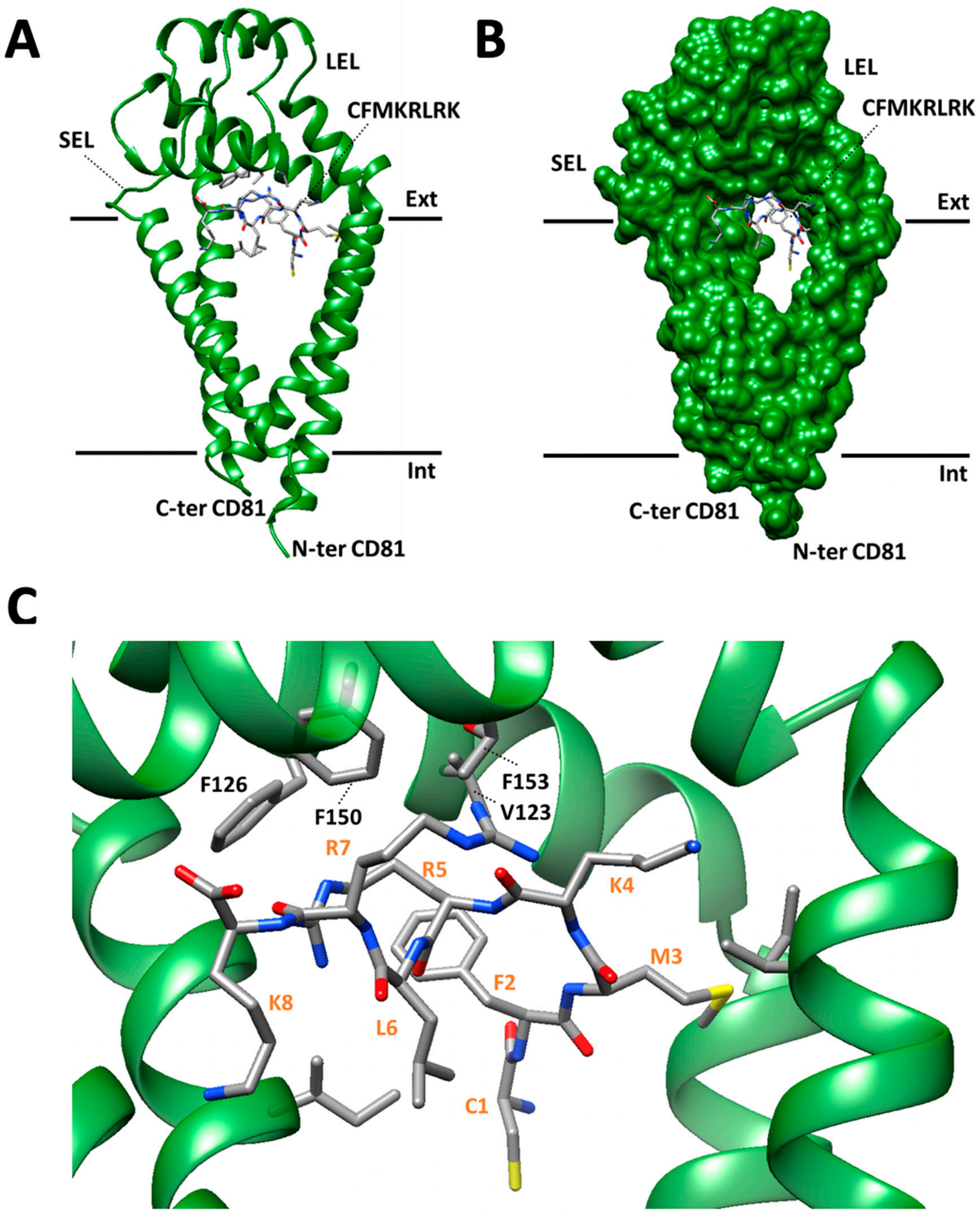

Figure S8. Protein−peptide complex structure of P152 and CD81 protein predicted by MDockPeP server [22]. (A) Protein−peptide complex structure of P152 and CD81 protein. (B) Surface representation of the molecular docking. (C) Zoomed view of the molecular docking. LEL, large extracellular loop. SEL, small extracellular loop. Ext, extracellular. Int, intracellular.

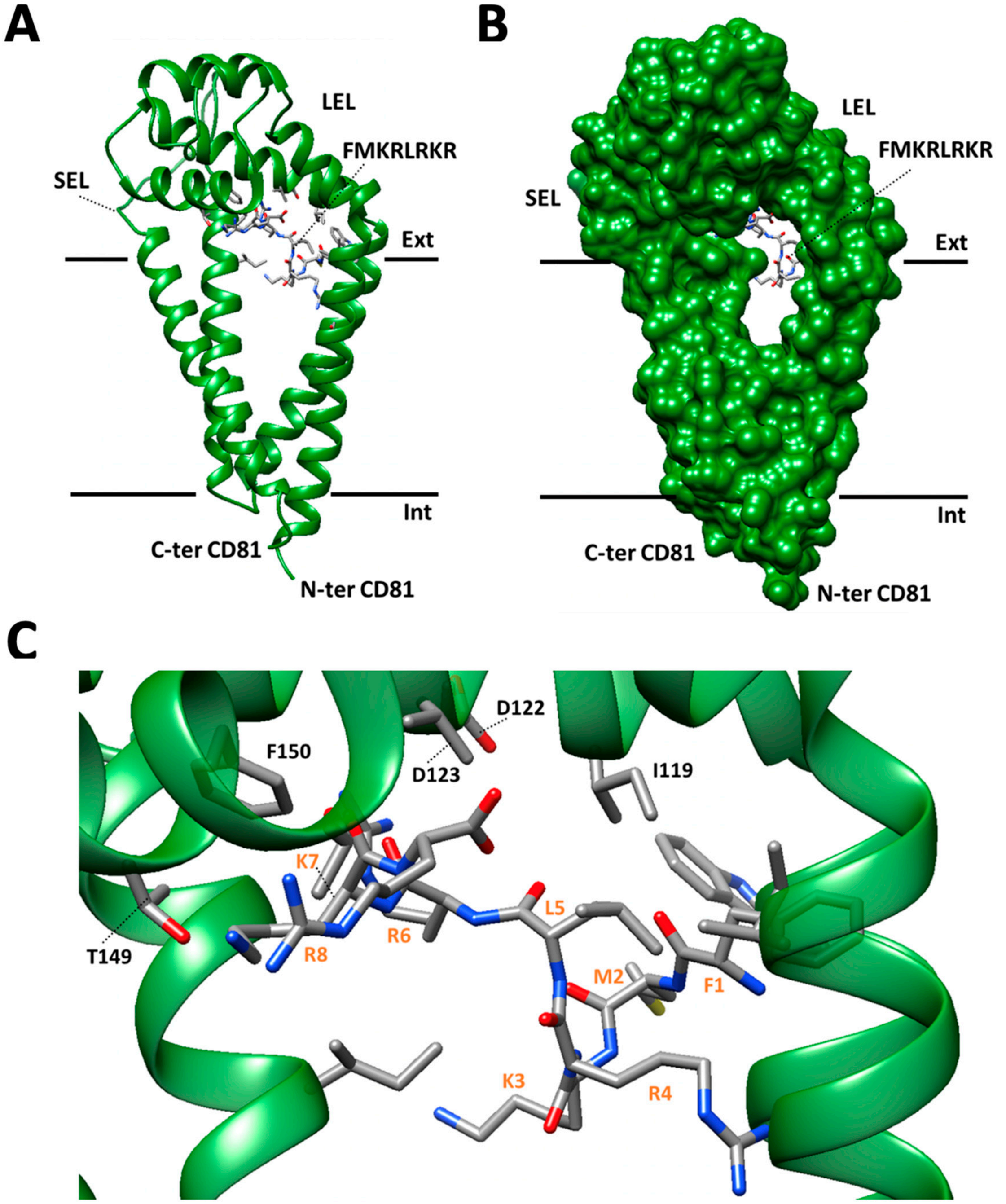

Figure S9. Protein−peptide complex structure of P153 and CD81 protein predicted by MDockPeP server [22]. (A) Protein−peptide complex structure of P153 and CD81 protein. (B) Surface representation of the molecular docking. (C) Zoomed view of the molecular docking. LEL, large extracellular loop. SEL, small extracellular loop. Ext, extracellular. Int, intracellular.

Table S2. Docking energy scores of P152 for CD81, EPCAM, and CD44 assessed by HPEPDOCK: a web server for blind peptide-protein docking based on the hierarchical algorithm [23].

Peptide	Protein	Docking Energy Score (-)
P152	CD81	-181.751
P152	EpCAM	-164.651
P152	CD44	-162.779

Table S3. Docking energy scores of P152 and its scrambled sequences for CD81 assessed by HPEPDOCK: a web server for blind peptide-protein docking based on the hierarchical algorithm [23].

Peptide	Sequence [a]	Docking energy score (-)
P152	CFMKRLRK	-181.751
P152-1	FRKCKMRL	-164.0808
P152-2	KKRCRFLM	-165.9216
P152-3	RFMRCLKK	-166.6464

[a] Sequence of three scrambled peptides from P152 (P152-1, P152-2 and P152-3) was generated by an unbiased and auto sequence generator software (http://www.mimotopes.com/ peptideLibraryScreening.asp?id=97).

A

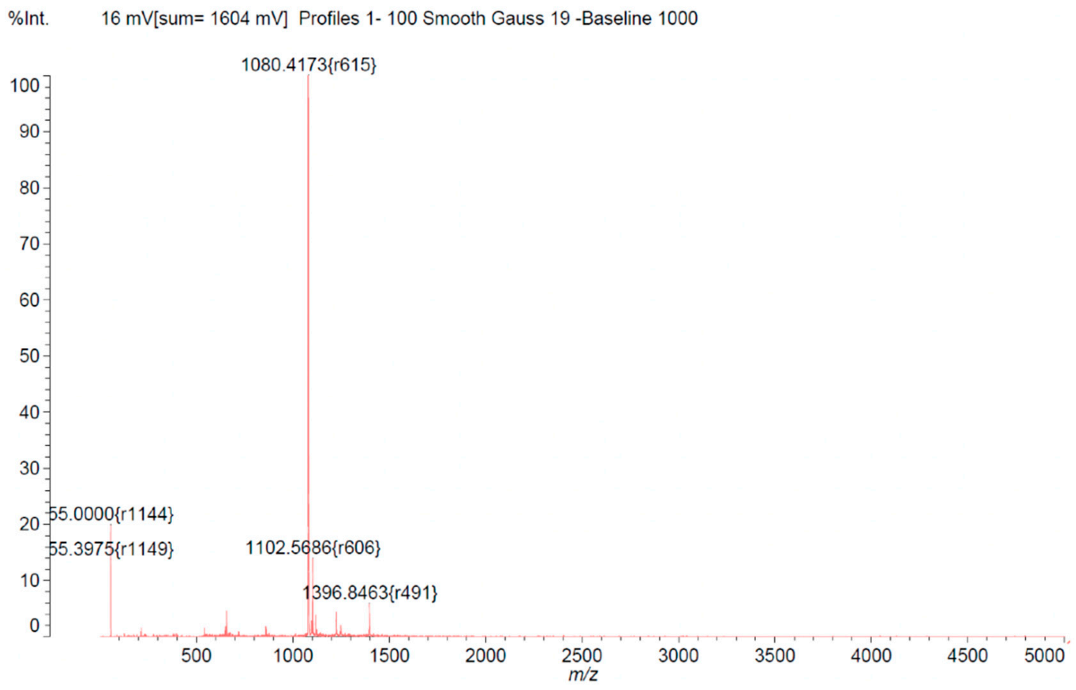

B

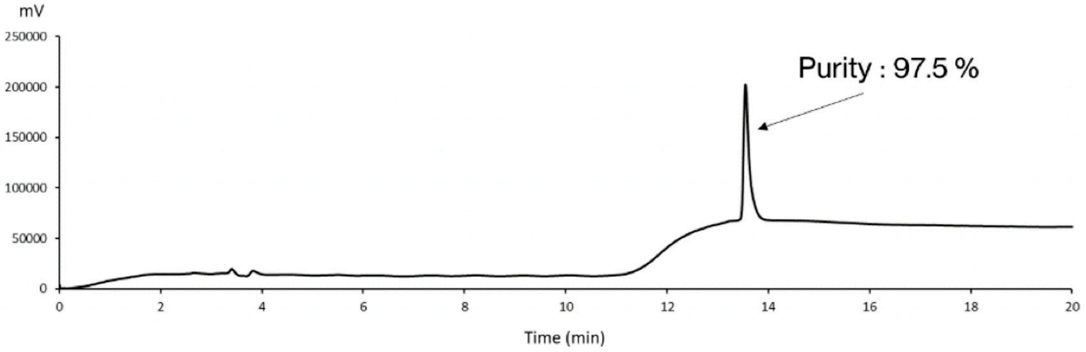

Figure S10. (A) MS spectrogram and (B) HPLC chromatogram of P152-1.

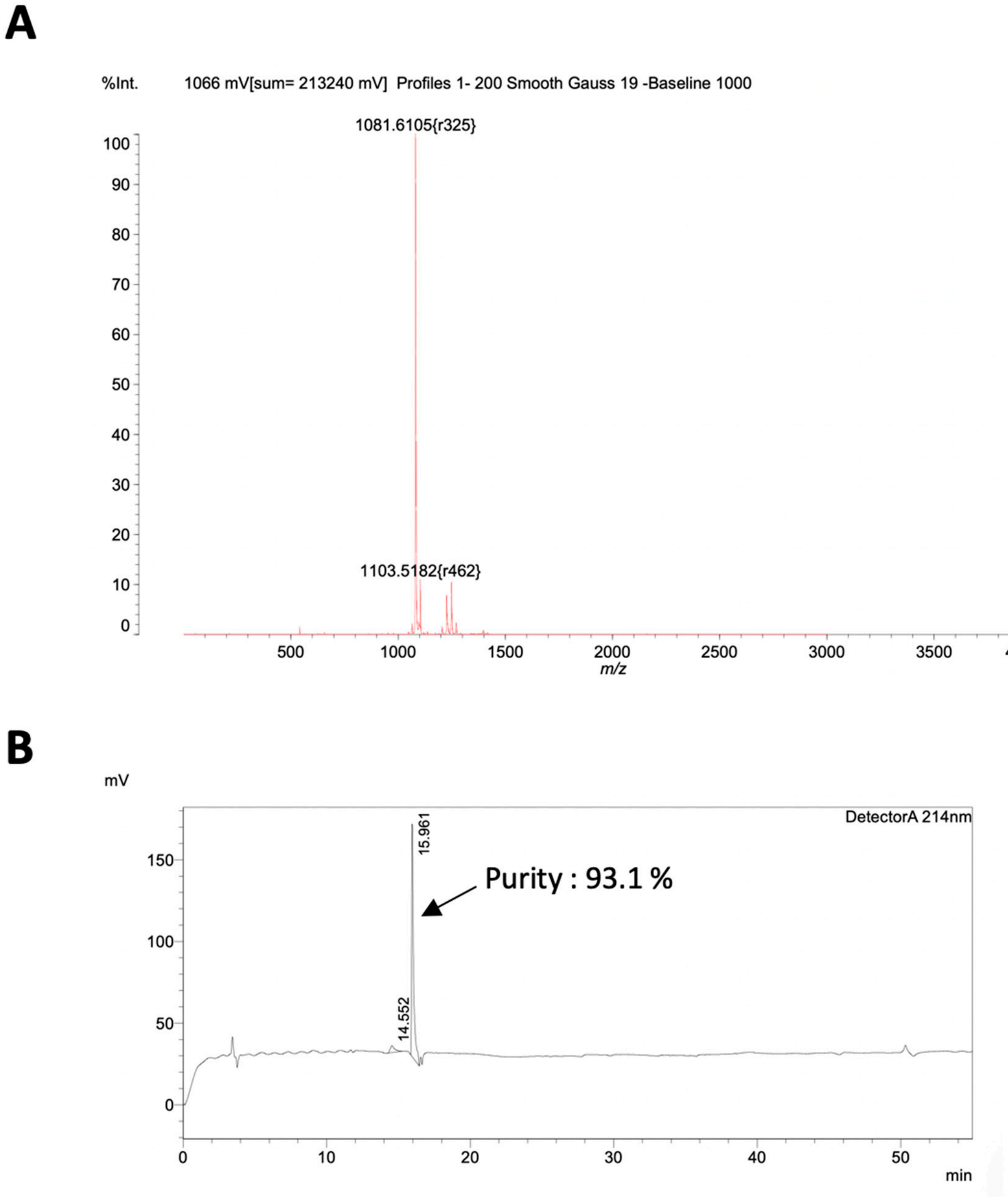

Figure S11. (A) MS spectrogram and (B) HPLC chromatogram of P152-2.
